# Luminescent Cd coordination polymer based on thiazole as a dual-responsive chemosensor for 4-nitroaniline and CrO_4_^2−^ in water

**DOI:** 10.1038/s41598-023-27466-x

**Published:** 2023-01-06

**Authors:** Akram Karbalaee Hosseini, Azadeh Tadjarodi

**Affiliations:** Research Laboratory of Inorganic Materials Synthesis, Department of Chemistry, Iran University of Science and Technology (IUST), Tehran, 16846-13114 Iran

**Keywords:** Chemistry, Coordination chemistry

## Abstract

A novel highly fluorescent cadmium metal–organic framework, [Cd (DPTTZ) (OBA)] (IUST-3), synthesized by using two linkers 2, 5-di (pyridine-4-yl) thiazolo [5, 4-d] thiazole (DPTTZ) and 4, 4′-oxybis (benzoic acid) (OBA) simultaneously, which exhibits a two-dimensional framework. The characteristics of this Cd-MOF were investigated by single-crystal X-ray diffraction, Fourier transform infrared spectroscopy, elemental analysis, powder X-ray diffraction, and thermogravimetry analysis. The IUST-3 exhibits excellent luminescence property and good stability in water. Luminescent experiments indicate that the IUST-3 has remarkable sensitivity and selectivity for the detection of 4-nitroaniline (4-NA), and CrO_4_^2−^ anion with *K*_SV_ = 1.03 × 10^5^ M^-1^ (4-NA) and *K*_SV_ = 2.93 × 10^4^ M^-1^ (CrO_4_^2−^) and low limit of detection 0.52 µM (4-NA) and 1.37 µM (CrO_4_^2−^). In addition, the possible fluorescence quenching mechanism was explored in this paper.

## Introduction

Sensation and detection of pollution in the water environment are essential to environmental conservation and life safety. Wastewater generated by various industrial processes such as alloying, electroplating, corrosion inhibition, printing, textile dyeing, etc., contains chromium and its derivatives^1–4^. Hexavalent chromium compound, CrO_4_^2^ anion, is very dangerous for human health that can cause serious adverse health effects, such as kidney damage, skin irritation, lung carcinoma, allergic reactions, and so on^5–7^. In addition, 4-nitroaniline, a well-known explosive compound, as an essential intermediate in the synthesis of pesticides chemicals, dyes, photostabilizers, antioxidants, and pharmaceuticals has become a serious pollution source of soil, groundwater, and sediments^8–10^. Therefore, potentially cause some severe diseases, such as cancer, coma, anemia, methemoglobinemia, and so on^11–13^. Thus, the expansion of extremely efficient methods to detect hazardous substances in the water environment has become increasingly necessary and attracted considerable research attention^14–17^.

Metal–organic frameworks (MOFs), also known as porous coordination polymers (PCPs), are a fascinating type of sensory materials self-assembled by metal ions/clusters and organic bridging linkers/ligands via coordination bonds, which incorporate the inherent merits of the rigid inorganic materials and flexible organic materials. In the realm of metal–organic frameworks, luminescent MOFs (LMOFs) have drawn tremendous attention over the last two decades from both scientific researchers and industrial engineers^18^.

Suitable material for use as chemical sensors can be divided into multiple classes based on the kind of feature, which leads to a detection signal. In the case of optical sensors, detection is attained by a change in color, fluorescence, or circular dichroism of the sensor element. Analytical techniques based on luminescence due to the high efficiency with which light can lead to single-molecule detection and recent advances in detector and optical technologies have attracted significant attention. Among multiple luminescent materials, LMOFs newly appear as potential chemical sensors because of their easily induced luminescence, various advantages in structural and functional ingredients, and diverse detecting mechanisms^19–23^.

Incorporating a bicyclic aromatic thiazolo[5,4-d]thiazole as an important type of multifunctional heterocyclic compound into the framework could lead to LMOFs with potential sensory properties^24–26^. With this idea in mind, we have designed a novel MOF, [Cd (DPTTZ) (OBA)] (IUST-3), containing thiazolo[5,4-*d*] thiazole unit as a luminescent metal–organic framework. The IUST-3 shows great fluorescent properties because of having Cd(II) cation with d^10^ electronic configuration and the strong *π*-conjugated effect of 2, 5-di (pyridine-4-yl) thiazolo [5, 4-d] thiazole ligand. In this work, we reported the synthesis, and crystal structure of the IUST-3, and also the selective detection of 4-nitroaniline (4-NA), and chromate anion (CrO_4_^2−^) in aqueous solution at room temperature by the IUST-3.

## Experimental

### Materials and general methods

All starting reagents were purchased from commercial suppliers and were used without further purification. The compound 2, 5-di (pyridine-4-yl) thiazolo [5, 4-d] thiazole (DPTTZ) as a ligand was synthesized as reported in the literature^27^. Infrared spectra were recorded in the range of 400–4000 cm^−1^ with a Shimaduz FT-IR-8400 spectrometer from samples in KBr pellets. Elemental analyses (CHNS) were done using a CHNS Thermo Finnigan Flash 1112 series elemental analyser. Powder X-ray diffraction measurements were performed using a Philips X’pert diffractometer. Thermo gravimetric analysis (TGA) was performed with Perkin Elmer Pyris 1 thermo gravimeter under argon (Ar) atmosphere in the range from 50 to 800 °C with a ramp rate of 10 °C min^−1^. The fluorescent spectra were recorded on a PerkinElmer LS45 fluorescence spectrometer.

### X-ray crystal structure determination

X-ray single-crystal data collection for the IUST-3 was performed at 296 K using Mo Kα radiation (λ = 0.71073 Å). Computing details include Data collection: MAR345 dtb Program (1.24–4, 2013); cell refinement: Automar software package (3.3a, 2015); data reduction: Automar software package (3.3a, 2015); program(s) used to solve structure: *SHELXT* 2018/2 (Sheldrick, 2018); program(s) used to refine structure: *SHELXL2016*/6 (Sheldrick, 2016); molecular graphics: *DIAMOND* (Brandenburg, 1999); software used to prepare material for publication: *PLATON* (2018). Table S1 summarized single-crystal X-ray diffraction data and structure refinement for the IUST-3.

### Synthesis of [Cd (DPTTZ) (OBA)] (IUST-3)

Cd(NO_3_)_2_ 4H_2_O (0.21 g, 0.67 mmol), DPTZTZ (0.10 g, 0.34 mmol), and 4,4′-oxybis(benzoic acid) (OBA) (0.17 g, 0.67 mmol) were dissolved in 50 mL of DMF, and then the solution was placed in an 80 mL glass vial. The mixture was heated at 95 °C for 72 h. After slow cooling to room temperature, yellow single crystals were formed and collected by filtration and then washed with hot DMF several times. Yield: 52% (based on DPTTZ). Elemental analysis (%), Cal. for C_28_H_16_CdN_4_O_5_S_2_: C, 50.57; H, 2.43; N, 8.43; S, 9.64, found: C, 51.02; H, 2.86; N, 8.84; S, 9.13. IR (KBr, cm^-1^): 3422(m), 3058(m), 2924(m), 2853(w), 1676(s), 1596(s), 1540(m), 1500(m), 1392(s), 1240(s), 1159(s), 1093(m), 1064(m), 1011(s), 880(s), 833(m), 783(s), 702(s), 660(s), 618(s), 506 (s), 411(m).

### The luminescent experiments

The photoluminescence properties of the IUST-3 were investigated in H_2_O suspensions at room temperature. These suspensions were prepared by adding 1 mg of the IUST-3 powder into 10 mL of H_2_O and dispersed under 20 min ultrasonic irradiation (60 W) before testing.

For selectivity CrO_4_^2−^ anion and 4-NA (1 × 10^−3^ M, 100 μM) were added to 2 mL of the IUST-3 suspension, containing different anions and aromatic compounds (1 × 10^−3^ M, 100 μM), respectively. Then after 1 min, the fluorescence spectra were measured upon excitation at 380 nm.

In the fluorescence titration experiment, 2 mL of the IUST-3 suspension was added to a quartz cell, and the photoluminescence spectra (excitations at 380 nm) were recorded after each incremental addition of 20 µL and 10 µL analyte solution (1 × 10^−3^ M) for CrO_4_^2−^ anion and 4-NA, respectively.

## Results and discussion

### Characterization of [Cd (DPTTZ) (OBA)] (IUST-3)

#### Crystal structure

**Figure 1:**
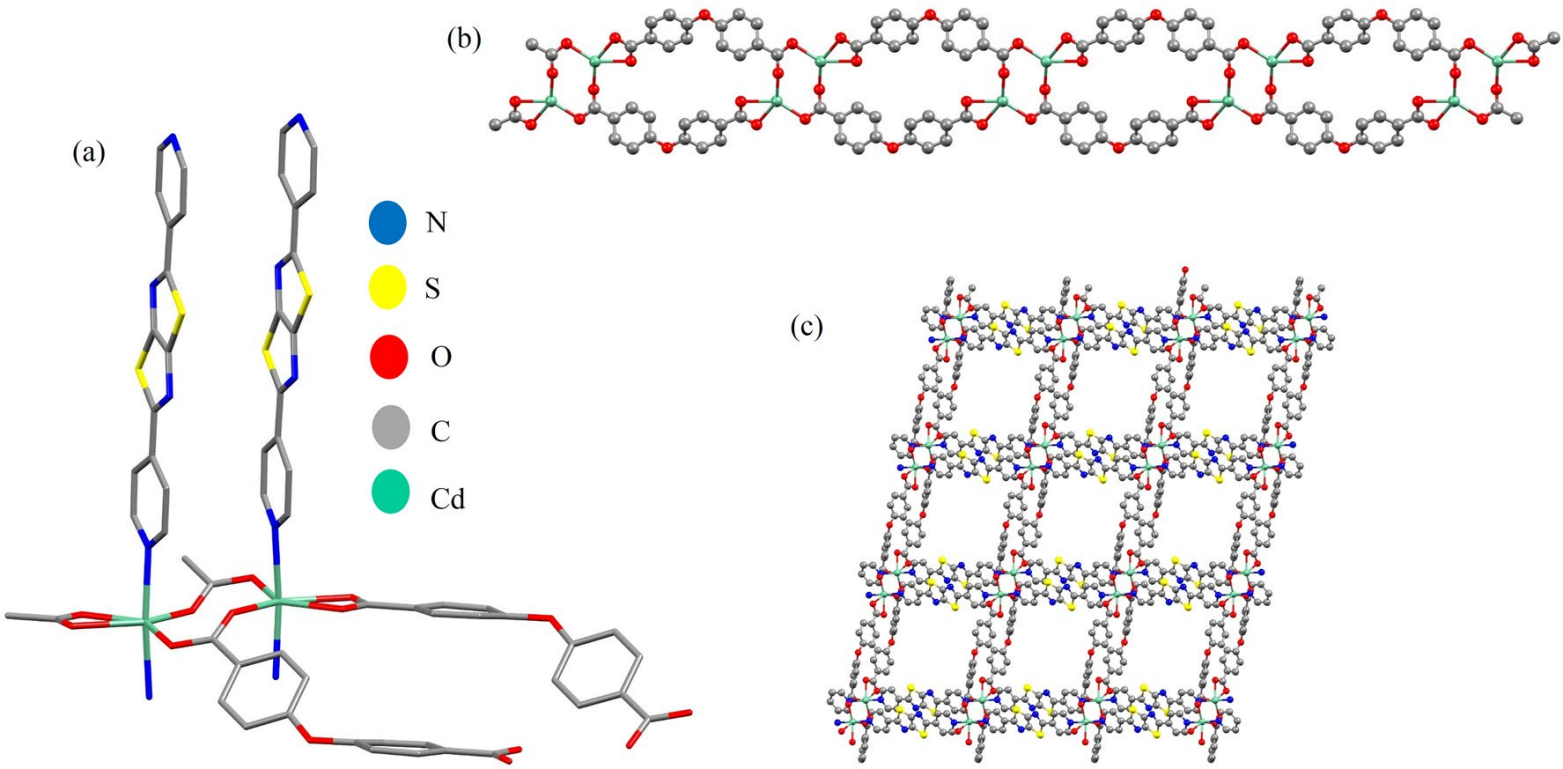
(**a**) View of coordination environment of the Cd(II) ions in the IUST-3; Symmetry codes: (i) − *x*, − *y* + 2, − *z*; (ii) − *x*, − *y* + 2, − *z* + 1; (iii) *x* − 1, *y* + 1, *z*; (iv) *x* + 1, *y* − 1, *z*, (**b**) 1D double-chain of Cd(II) ions and OBA ligands, (**c**) 2D pillared-layer framework of the IUST-3.

**Figure 2:**
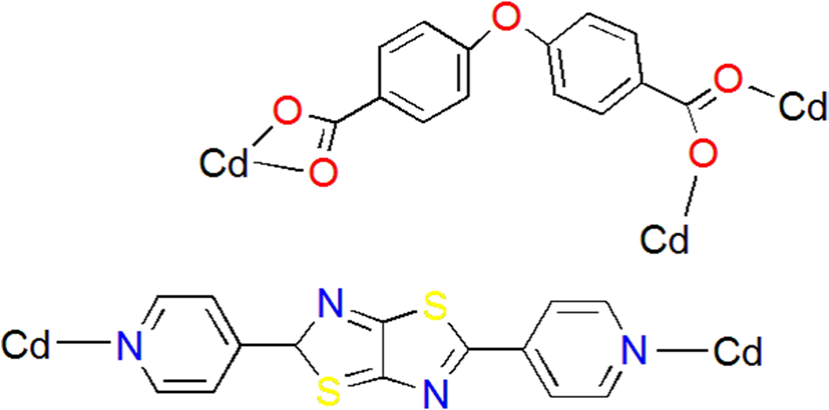
Coordination modes of the DPTTZ and OBA ligands in the IUST-3.

The IUST-3 is composed of Cd(II) metal nodes linked by DPTTZ and OBA linkers and represents a 2D framework, which crystallizes in the triclinic crystal system with space group $P\overline{1 }$. The asymmetric unit of the IUST-3 consists of one Cd(II) center, one DPTZTZ ligand, and one OBA ligand. The Cd(II) atom of the IUST-3 is six-coordinated displaying a distorted octahedral coordination environment by two nitrogen atoms from two different 2, 5-di (pyridine-4-yl) thiazolo [5, 4-d] thiazole (DPTTZ) ligands and four oxygen atoms from two different 4,4′-oxybis(benzoic acid) (OBA) ligands. Four oxygen atoms from OBA ligands together with the central Cd(II) ion form the equatorial plane and two nitrogen atoms from DPTZTZ ligands occupy the axial positions (Fig. 1a). The Cd–N bond lengths are 2.351(5) Å and 2.371(4) Å, and the Cd–O bond distances are in the range of 2.270(5) to 2.403(4) Å. The O–Cd–O and N–Cd–O angles are in the range of 54.72(14)–147.38(14)° and 84.61(16)–96.49(16)°, respectively. The two crystallographically independent Cd(II) ions also form a dimeric secondary building unit (SBU) Cd_2_(μ-OCO)_2_. The adjacent dimeric SBUs are further bridged by OBA ligands, whose carboxyl groups adopt μ_1_-η^1^:η^1^-bidentate chelating and μ_2_-η^1^:η^1^-bismonodentate bridging mixed mode (Fig. 2), providing a 1D double-chain {Cd_2_(OBA)_2_}_*n*_ along the *ac* plane (Fig. 1b). Further, the adjacent double-chains are expanded by DPTTZ ligands to construct a 2D sheet along the *bc* plane (Fig. 1c).

#### PXRD, FT-IR, and thermal property

To confirm the phase purity of the IUST-3, the powder X-ray diffraction (PXRD) was carried out. The result showed that the PXRD pattern of as-synthesized the IUST-3 is in good accordance with the simulated pattern from its crystal data. Considering the practical application of the IUST-3 as a sensor for detection of anions and aromatic compounds in aqueous media, the water stability of the IUST-3 was further studied. The results of the PXRD before and after the immersion process in water revealed that the IUST-3 remain constant after the immersion process (Fig. S1).

Figure S2 shows the FT-IR spectrum of the IUST-3. Dicarboxylate groups of 4,4′-oxybis (benzoic acid) ligands show characteristic absorption bands at 1540 cm^−1^ and 1443 cm^−1^ for asymmetric and symmetric, respectively^28^. As all carboxyl groups of 4,4′-oxybis (benzoic acid) ligands in the reaction with Cd ions have deprotonated, there is no absorption band at 1700 cm^−1^ for protonated carboxylate groups. The peak at 1675 cm^−1^ is attributed to the carbonyl group of DMF molecules^29^. The band in the 3425 cm^−1^ is probably attributed to the presence of water in the KBr matrix. The relatively weak absorption band at 3056 cm^−1^ results from the C–H modes involving aromatic rings.

The TGA curve shows that the IUST-3 is thermally stable under Ar stream up to 340 °C (Fig. S3). The IUST-3 demonstrates a one-stage weight loss process, loss of mixed ligands from 340 °C, which was the main thermal loss of 55.2%. The remaining weight may be attributed to the formation of cadmium oxide, cadmium sulfide, cadmium sulfate, or a mixture of these compounds.

#### Sensing of aromatic compounds

**Figure 3:**
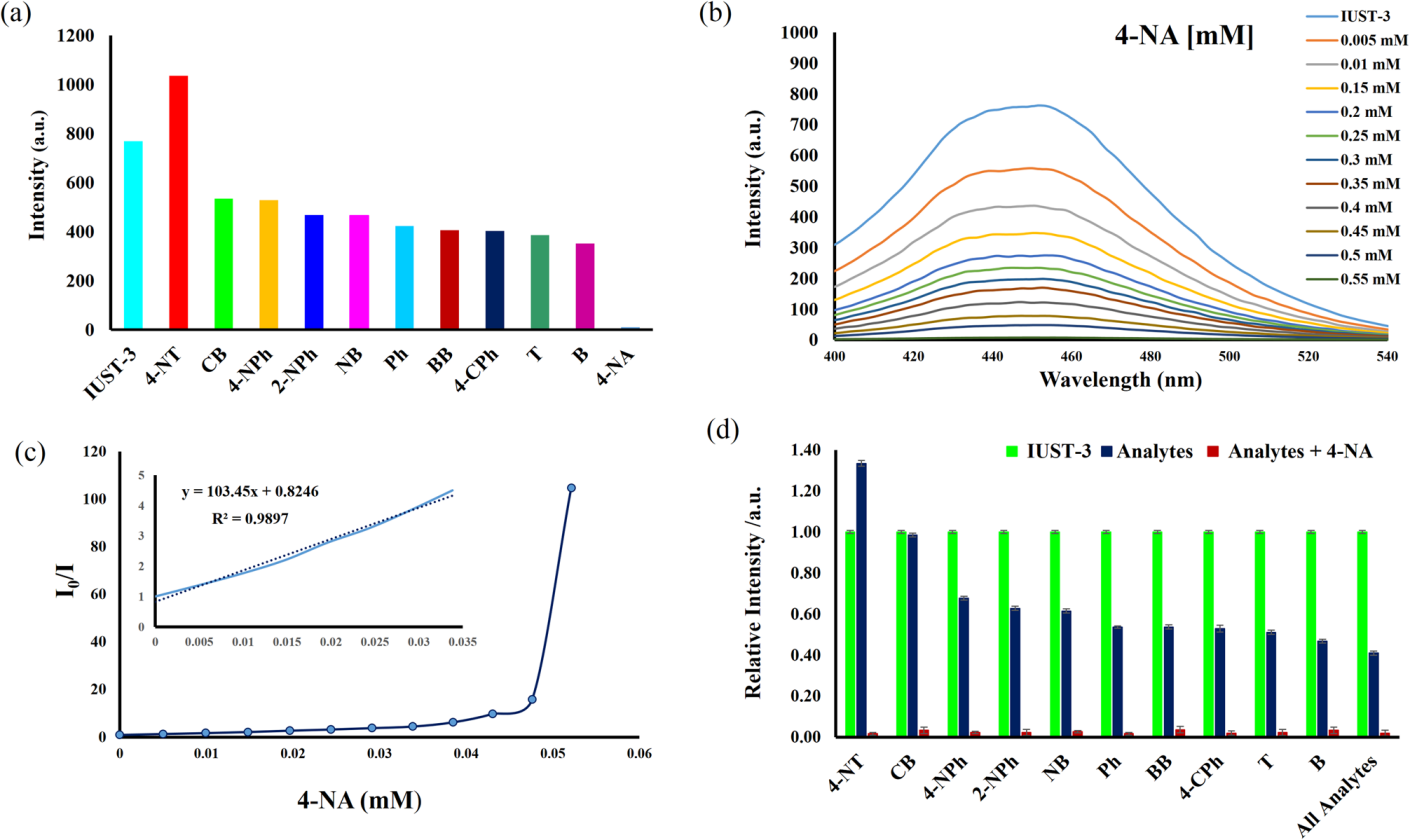
(**a**) The comparison of the fluorescence quenching intensity of the IUST-3 (1 mg) in water (2 ml) among the aromatic compounds (1 mM, 100 µl), (**b**) Photoluminescence spectra of the IUST-3 with gradual addition of 4-NA (1 mM, 10 µL addition each time), (**c**) Stern–Volmer plot of I_0_/I versus 4-NA concentration in the IUST-3 aqueous suspension (insert: enlarged view of a selected area), (**d**) Selective detection of 4-NA on the IUST-3 in the presence of different aromatic compounds in water.

Aromatic compounds (ACs) are a class of undesirable organic pollutants with a benzene ring in wastewater and can cause fluorescence quenching in porous luminescent MOFs due to electron or energy transfer^30–32^. Considering the excellent luminescence properties of the IUST-3 in aqueous solution, the ability of the IUST-3 to detect aromatic compounds in aqueous media through luminescent sensing has been investigated. Therefore, a diversity of aromatics compounds with and without nitro groups were selected including 2-nitrophenol (2-NPh), 4-nitrophenol (4-NPh), 4-nitroaniline (4-NA), 4-nitrotoluene (4-NT), nitrobenzene (NB), benzene (B), toluene (T), phenol (Ph), bromobenzene (BB), chlorobenzene (CB), 4-chlorophenol (4-CPh). The quantitative sensitization determination of quenching behavior was performed by the addition of aromatic compounds (1 × 10^−3^ M, 100 µL) to the suspension of the IUST-3 (dispersing 1 mg of the IUST-3 in 10 mL of H_2_O) and the luminescence intensities were recorded. Fluorescence emission data were collected at *λ*_ex_ = 380 nm and emission wavelengths at 400 ~ 550 nm. As depicted in Fig. 3a, among the aromatic compounds, 4-nitroaniline displays the greatest quenching effect on the luminescence of the IUST-3. The photoluminescence intensity of the IUST-3 decreased more than 98% with only the addition of 110 μL 4-NA solution and the order of the quenching efficiency for different aromatic compounds was in the order, 4-NA > B > T > 4-CPh > BB > Ph > NB > 2-NPh > 4-NPh > CB > 4-NT.

A fluorescence titration experiment was carried out with gradual addition of 4-NA (1 × 10^−3^ M, 10 µL addition each time). As the 4-NA gradually increased, the luminescence intensity of the IUST-3 at 452 nm gradually decreased (Fig. 3b). The quenching degree can be quantitatively measured by *K*_SV_ (quenching effect constant) using the Stern–Volmer equation: *I*_*0*_/*I* = 1 + *K*_SV_ [M], where *I*_0_ and *I* are the fluorescence intensities before and after the additions of the analyte, respectively and [M] is the molar concentration of the analyte. The Stern–Volmer curve for 4-NA is linear at low concentrations. However, with the increase in concentration, the curve deviates from the linearity, which can be explained by an energy–transfer process or self-absorption^33^. As shown in Fig. 3c, the quenching constant *K*_SV_ of the IUST-3 for 4-NA was calculated to be 1.03 × 10^5^ M^−1^ based on the linear part, which is at a relatively high level than other LMOFs for sensing 4-NA (Table S2). The above results propose that the IUST-3 can act as a sensor for 4-NA in aqueous solutions with high sensitivity. The limit of detection (LOD) was calculated according to the equation LOD = 3*σ*/*k*, where *σ* represents the standard deviation of a blank sample and *k* is defined as the slope of the linear calibration plot. The LOD of the IUST-3 for 4-NA is 0.52 µM, which is among the lowest reported detection limits for the LMOFs (Table S2). The competing behavior for selective detection of 4-NA by the IUST-3 over the other analytes was carried out by registering the luminescence spectra of aqueous suspensions of the IUST-3 in the presence of 4-NA and different interfering aromatic compounds, where after the addition of (100 µL, 1 × 10^−3^ M) of the respective analyte, (100 µL, 1 × 10^−3^ M) of 4-NA was added (Fig. 3d). The quenching phenomenon of 4-NA on the IUST-3 is not changed even in presence of added different aromatic compounds, demonstrating that the IUST-3 can be considered as a potential luminescent probe to detect 4-NA among the above-mentioned different aromatic compounds.

**Figure 4:**
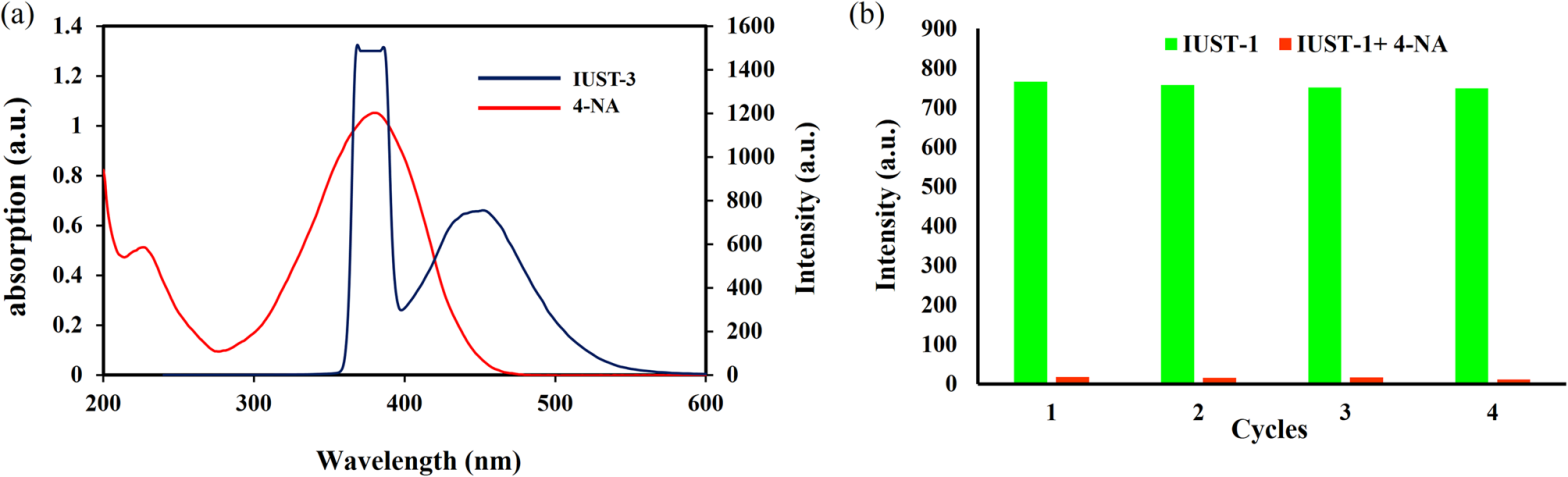
(**a**) Spectral overlap of absorbance spectrum of 4-NA with excitation and emission spectra of the IUST-3, (**b**) Luminescent intensity of the IUST-3 at 380 nm after four cycles for 4-NA.

The reason for the luminescence quenching caused by 4-NA is discussed as follows: the collapse of the framework, the photoinduced electron transfer (PET), and the resonance energy transfer (RET). The PXRD pattern of the IUST-3 after detecting 4-NA was measured, which is similar to the initial one (Fig. S4) suggesting that the fluorescence intensity reduction of the IUST-3 was not due to the decomposition of the framework structure. In the PET process, the LUMO of fluorescent ligand must have higher energy in comparison to the LUMOs of acceptor analytes. The conduction band of the DPTTZ ligand (LUMO = -2.665 eV) is located at higher energy than the LUMO of 4-NA (LUMO = − 2.816 eV), demonstrating the PET process is the reason for fluorescence intensity reduction but might be not the only mechanism for luminescence quenching for the IUST-3^34,35^. Therefore, considering the nonlinear behavior from the SV plot for 4-NA at higher concentrations, the resonance energy transfer (RET) is probably the other proposed mechanism for detecting 4-NA. As indicated in Fig. 4a, the absorption spectrum of 4-NA illustrated a strong overlap with the excitation and emission spectra of the IUST-3. According to these results, 4-NA can absorb the excitation and emission energy of the IUST-3, resulting in luminescence quenching^36,37^.

As a sensor, in addition to being stable, selective, and sensitive, regeneration is another significant issue. The reusability of the fluorescence sensing performances of the IUST-3 towards 4-NA was examined for up to four cycles of detection. The recyclability of the IUST-3 in the sensing process was evaluated. We dispersed the IUST-3 in the H_2_O solution (1 × 10^−3^ M) of 4-NA to generate the IUST-3@4-NA sample. The resulting solid was treated several times with water to entirely wash 4-NA. As depicted in Fig. 4b, the fluorescence quenching ability of the untreated the IUST-3 is equivalent to the recovered the IUST-3 after four cycles, which indicates the IUST-3 is a recyclable luminescent probe for the CrO_4_^2−^ anion.

#### Sensing of anions

**Figure 5:**
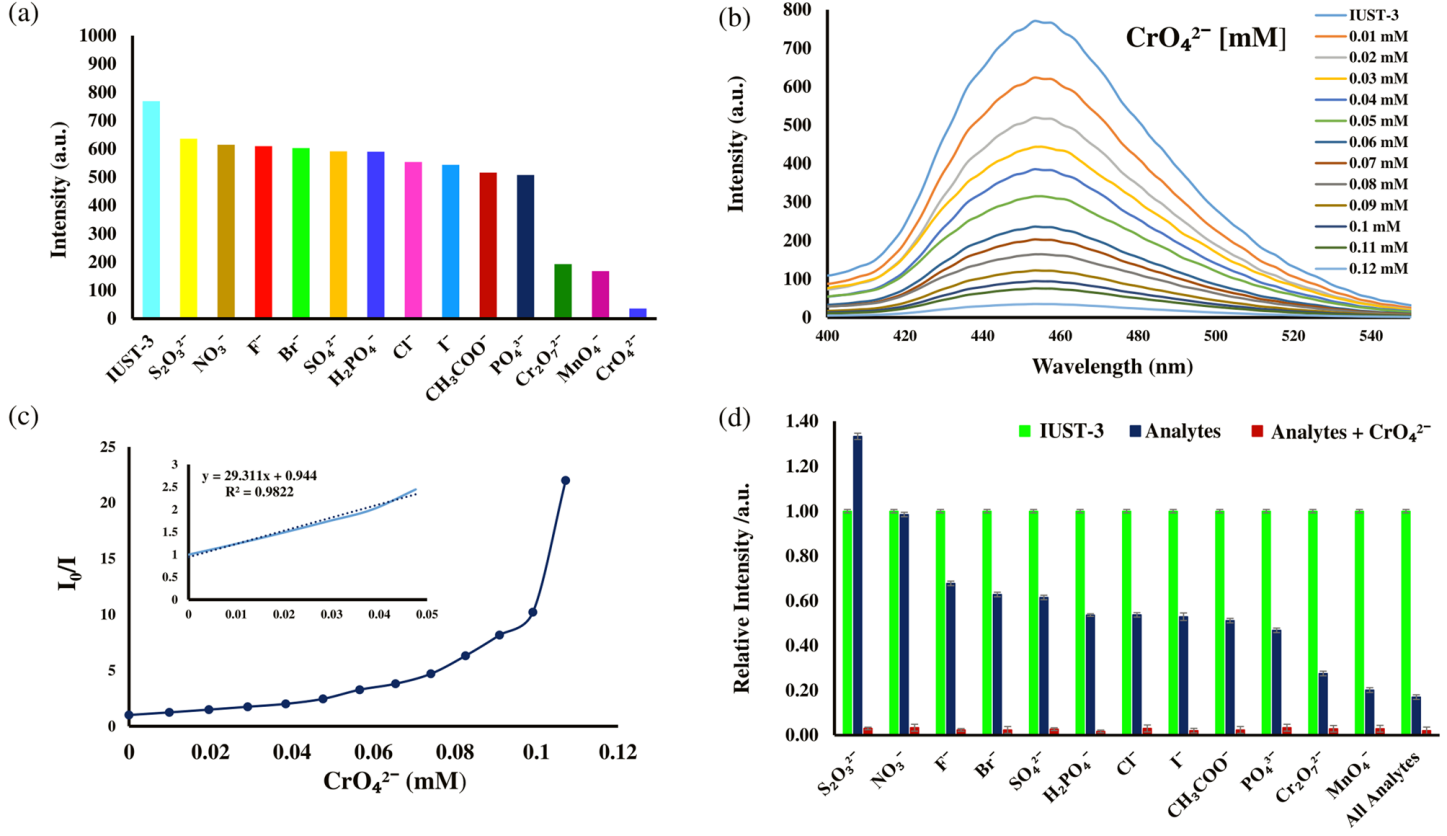
(**a**) The comparison of the fluorescence quenching intensity of the IUST-3 (1 mg) in water (2 ml) among the different anions (1 mM, 100 µl), (**b**) Photoluminescence spectra of the IUST-3 with gradual addition of the CrO_4_^2−^ anion (1 mM, 10 µL addition each time), (**c**) Stern–Volmer plot of I_0_/I versus the CrO_4_^2−^ anion concentration in the IUST-3 aqueous suspension (insert: enlarged view of a selected area), (d) Selective detection of the CrO_4_^2−^ anion on the IUST-3 in the presence of different aromatic compounds in water.

The quenching effect of the IUST-3 for the sensing of anions was investigated in KX aqueous solutions (X = I^−^, NO_3_^−^, SO_4_^2−^, Br^−^, Cl^−^, PO_4_^3−^, CH_3_COO^−^, F^−^, S_2_O_3_^2−^, H_2_PO_4_^−^, MnO_4_^−^, CrO_4_^2−^, and Cr_2_O_7_^2−^, 1 × 10^−3^ M). As depicted in Fig. 5a, different anions quench the luminescent intensity to a certain degree while the CrO_4_^2−^ anion almost completely quenches the fluorescence intensity of the IUST-3. The IUST-3 illustrates high quenching efficiency of 95.02% toward the CrO_4_^2−^ anion. The quenching efficiencies for the IUST-3 follow the order of CrO_4_^2−^ > MnO_4_^−^ > Cr_2_O_7_^2−^ > PO_4_^3−^ > CH_3_COO^−^ > I^−^ > Cl^−^ > H_2_PO_4_^−^ > SO_4_^2−^ > Br^−^ > F^−^ > NO_3_^−^ > S_2_O_3_^2−^. The quantitative sensitizations of the quenching behavior were also measured by changing the amount of CrO_4_^2−^ anion in the solution. With the increasing concentration of CrO_4_^2−^ anion, the emission intensities of the IUST-3 gradually decrease under excitation at 380 nm (Fig. 5b). With the increasing concentrations of CrO_4_^2−^ anion, the Stern–Volmer curve gradually deviated and bent upward (Fig. 5c). In the light of the linear fitting of the Stern–Volmer curve, the calculated K_*SV*_ value for CrO_4_^2−^ anion by the IUST-3 was 2.93 × 10^4^ M^-1^. In addition, the calculated detection limit for the CrO_4_^2−^ anion can be as low as 1.37 µM, which is comparable to the value obtained for the previously reported LMOFs for sensing the CrO_4_^2−^ anion (Table S3). Moreover, anti-interference experiments were performed in the presence of the CrO_4_^2−^ anion (100 µL, 1 × 10^−3^ M) and different anions (100 µL, 1 × 10^−3^ M). With the addition of the CrO_4_^2−^ anion, the luminescence intensity was significantly quenched (Fig. 5d). The above results suggest that the IUST-3 can serve as a good selective and sensitive “turn-off” fluorescent probe for the detection of CrO_4_^2−^ anion, which is not interfered by other anions.

**Figure 6:**
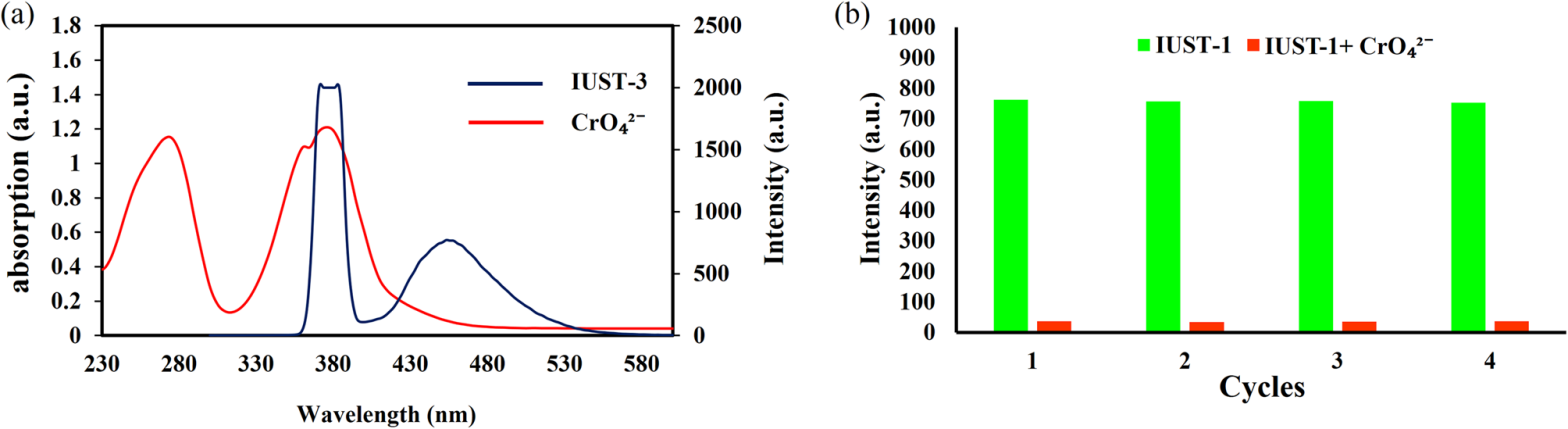
(**a**) Spectral overlap of absorbance spectrum of the CrO_4_^2−^ anion with excitation and emission spectra of the IUST-3, (**b**) Luminescent intensity of the IUST-3 at 380 nm after four cycles for the CrO_4_^2−^ anion.

The conceivable quenching mechanism of luminescence sensing for the CrO_4_^2−^ anion is perused. The PXRD patterns represent that the IUST-3 remains intact after detection (Fig. S4), which confirms that the decomposition of the IUST-3 was not a reason for luminescence quenching. To determine whether the IUST-3 and CrO_4_^2−^ interact, FT-IR spectra were collected before and after sensing experiments. The characteristic absorption peaks of the IUST-3 were not altered after adding CrO_4_^2−^, demonstrating that there is no coordination between CrO_4_^2−^ and the IUST-3 (Fig. S5). The absorption spectrum of the CrO_4_^2−^ anion showed a remarkable overlap with the excitation and emission spectra of the IUST-3 (Fig. 6a). This fact relates that the resonance energy transfer mechanism is operative in luminescence quenching, which demonstrates the good sensitivity and selectivity of the IUST-3 toward CrO_4_^2−^ anion^38^.

To demonstrate recyclability, the IUST-3 dispersed in the water solution (1 × 10^−3^ M) of CrO_4_^2−^ anion to generate the IUST-3@CrO_4_^2−^sample. The resulting solid was washed several times with water to entirely wash the CrO_4_^2−^ anion and then dried in the air. As shown in Fig. 6b, the fluorescence intensity of the recovered the IUST-3 remains essentially unchanged after four consecutive cycles, which represents its good stability and high reusability for the detection applications of the CrO_4_^2−^ anion.

## Conclusions

In summary, incorporating thiazolo[5,4-*d*] thiazole unit into a cadmium metal–organic framework lead to the fabrication of a new LMOF as a good dual-responsive sensor. A water-stable luminescent Cd-MOF (IUST-3) was successfully synthesized by the solvothermal reaction. Particularly, the IUST-3 can detect CrO_4_^2−^ anion and 4-nitroaniline (4-NA) in an aqueous solution with high sensitivity and selectivity through a fluorescence quenching effect. In addition, the cycling experiment reveals that the IUST-3 has good reusability for CrO_4_^2−^ anion and 4-nitroaniline (4-NA) detection with negligible changes in luminescent intensity after four cycles. There are also further discussions of the mechanism of the quenching effect and sensing properties of the IUST-3. The present results reveal that the use of the IUST-3 is a simple, rapid, selective, and non-expensive method for 4-nitroaniline and CrO_4_^2−^ anion sensing.

## Supplementary Information

Supplementary Information.

## Data Availability

All data generated or analyzed during this study are included in this published article [and its supplementary information files].

## References

[1] Han TU, Kim J, Kim K (2021). Accelerated chromate reduction by tea waste: Comparison of chromate reduction properties between water and ice systems. Environ. Res..

[2] Elwakeel KZ, Al-Bogami AS, Elgarahy AM (2018). Efficient retention of chromate from industrial wastewater onto a green magnetic polymer based on shrimp peels. J. Polym. Environ..

[3] El-Ashtoukhy ES, Abdel-Aziz MH, Sedahmed GH (2018). Simultaneous removal of greases and hexavalent chromium from electroplating and chromate conversion coating waste solution by electrocoagulation. Water Air Soil Pollut..

[4] Chang LY (2005). Chromate reduction in wastewater at different pH levels using thin iron wires—a laboratory study. Environ. Prog..

[5] Yu H (2020). Two highly water-stable imidazole-based Ln-MOFs for sensing Fe^3+^, Cr_2_O_7_^2^^−^/CrO_4_^2^^−^ in a water environment. Inorg. Chem..

[6] Liu J, Ji G, Xiao J, Liu Z (2017). Ultrastable 1D europium complex for simultaneous and quantitative sensing of Cr(III) and Cr(VI) ions in aqueous solution with high selectivity and sensitivity. Inorg. Chem..

[7] Chen S, Shi Z, Qin L, Jia H, Zheng H (2017). Two new luminescent Cd(II)-metal–organic frameworks as bifunctional chemosensors for detection of cations Fe^3+^, anions CrO_4_^2^^−^, and Cr_2_O_7_^2^^−^ in aqueous solution. Cryst. Growth Des..

[8] Yamuna A, Jiang TY, Chen SM (2021). Preparation of K^+^ intercalated MnO_2_-rGO composite for the electrochemical detection of nitroaniline in industrial wastewater. J. Hazard. Mater..

[9] Laghrib F, Farahi A, Bakasse M, Lahrich S, El Mhammedi MA (2019). Voltammetric determination of nitro compound 4-nitroaniline in aqueous medium at chitosan gelified modified carbon paste electrode (CS@CPE). Int. J. Biol. Macromol..

[10] Silambarasan S, Cornejo P, Vangnai AS (2022). Biodegradation of 4-nitroaniline by novel isolate *Bacillus* sp. strain AVPP64 in the presence of pesticides. Environ. Pollut..

[11] Qian L (2019). A ketone-functionalized carbazolic porous organic framework for sensitive fluorometric determination of p-nitroaniline. Mikrochim. Acta..

[12] Wei F (2018). A 1, 2, 3-triazolyl based conjugated microporous polymer for sensitive detection of p-nitroaniline and Au nanoparticle immobilization. Polym. Chem..

[13] Das P, Chakraborty G, Mandal SK (2020). Comprehensive structural and microscopic characterization of an azine–triazine-functionalized highly crystalline covalent organic framework and its selective detection of dichloran and 4-Nitroaniline. ACS Appl. Mater. Interfaces..

[14] Dubey M (2022). ZnO/α-MnO_2_ hybrid 1D nanostructure-based sensor for point-of-care monitoring of chlorinated phenol in drinking water. Mater. Today Chem..

[15] Li J (2021). A Cd-MOF fluorescence sensor with dual functional sites for efficient detection of metal ions in multifarious water environments. CrystEngComm.

[16] Xing P (2019). A Cd-MOF as a fluorescent probe for highly selective, sensitive and stable detection of antibiotics in water. Analyst..

[17] Pawar S, Kaja S, Nag A (2020). Red-emitting carbon dots as a dual sensor for In^3+^ and Pd^2+^ in water. ACS Omega.

[18] Wang Y-N (2022). A dual-chemosensor based on Ni-CP: Fluorescence turn-on sensing toward ascorbic acid and turn-off sensing toward acetylacetone. J. Lumin..

[19] Rozenberga L (2022). A europium metal–organic framework for dual Fe^3+^ ion and pH sensing. Sci. Rep..

[20] Wang Y-N (2022). Dual-responsive luminescent sensitivities of a 3D Co-CP with turn-on and ratiometric sensing toward ascorbic acid and turn-off detecting acetylacetone. J. Solid State Chem..

[21] Wang S-D (2020). A dual luminescent sensor coordination polymer for simultaneous determination of ascorbic acid and tryptophan. Spectrochim. Acta A Mol. Biomol. Spectrosc..

[22] Wang Y-N (2023). A water-stable dual-responsive Cd-CP for fluorometric recognition of hypochlorite and acetylacetone in aqueous media. Spectrochim Acta A Mol Biomol Spectrosc..

[23] Zhou Z (2018). A multifunctional nanocage-based MOF with tri- and tetranuclear zinc cluster secondary building units. Sci. Rep..

[24] Li P (2019). Interpenetration-enabled photochromism and fluorescence photomodulation in a metal–organic framework with the thiazolothiazole extended viologen fluorophore. Inorg. Chem..

[25] Kumar V (2022). Thiazolothiazole based donor-π-acceptor fluorophore: Protonation/deprotonation triggered molecular switch, sensing and bio-imaging applications. Anal. Chim. Acta..

[26] Dou Y (2022). Teaching a fluorophore new tricks: Exploiting the light-driven organic oxidase nanozyme properties of thiazolothiazole for highly sensitive biomedical detection. Sens. Actuators B Chem..

[27] Hosseini AK, Tadjarodi A (2022). Sonochemical synthesis of nanoparticles of Cd metal organic framework based on thiazole ligand as a new precursor for fabrication of cadmium sulfate nanoparticles. Mater. Lett..

[28] Rad FA, Rezvani Z (2015). Preparation of cubane-1,4-dicarboxylate–Zn–Al layered double hydroxide nanohybrid: Comparison of structural and optical properties between experimental and calculated results. RSC Adv..

[29] Hosseini AK, Pourshirzad Y, Tadjarodi A (2023). A water-stable luminescent cadmium-thiazole metal-organic framework for detection of some anionic and aromatic pollutants. J. Solid State Chem..

[30] Zhang Y (2020). A Cu(I)–I coordination polymer fluorescent chemosensor with amino-rich sites for nitro aromatic compound (NAC) detection in water. CrystEngComm.

[31] Jiang QL (2021). Luminescent zinc(II) coordination polymers of bis (pyridin-4-yl) benzothiadiazole and aromatic polycarboxylates for highly selective detection of Fe(III) and high-valent oxyanions. Cryst. Growth Des..

[32] Chen SS (2021). A photoluminescent Cd(II) coordination polymer with potential active sites exhibiting multiresponsive fluorescence sensing for trace amounts of NACs and Fe^3+^ and Al^3+^ ions. Inorg. Chem..

[33] Salinas Y (2021). Optical chemosensors and reagents to detect explosives. Chem. Soc. Rev..

[34] Li B, Yan QQ, Yong GP (2020). A new porous coordination polymer reveals selective sensing of Fe^3+^, Cr_2_O_7_^2−^, CrO_4_
^2−^, MnO_4_^−^ and nitrobenzene, and stimuli-responsive luminescence color conversions. J. Mater. Chem. C.

[35] Parmar B, Bisht KK, Rachuri Y, Suresh E (2020). Zn (II)/Cd (II) based mixed ligand coordination polymers as fluorosensors for aqueous phase detection of hazardous pollutants. Inorg. Chem. Front..

[36] Zhou AM, Wei H, Gao W, Liu JP, Zhang XM (2019). Two 2D multiresponsive luminescence coordination polymers for selective sensing of Fe^3+^, Cr(VI) anions and TNP in aqueous medium. CrystEngComm.

[37] Abdollahi N, Morsali A (2019). Highly sensitive fluorescent metal-organic framework as a selective sensor of Mn(VII) and Cr(VI) anions (MnO_4_^−^/Cr_2_O_7_^2−^/CrO_4_^2−^) in aqueous solutions. Anal. Chim. Acta..

[38] Wang ZY, Liu YR, Duan YL, Zhou R, Zhang X (2022). Manganese (II)-based coordination polymer as a bi-responsive luminescent sensor for highly selective detection of picric acid and CrO_4_^2−^ ion. Transit. Met. Chem..

