# Supplementary material for: Luminescent Cd coordination polymer based on thiazole as a dual-responsive chemosensor for 4-nitroaniline and CrO42− in water
Source: Sci Rep. 2023 Jan 6;13:269. doi: 10.1038/s41598-023-27466-x (PMC9822996; doi:10.1038/s41598-023-27466-x)
Supplement: Supplementary file 1 — Supplementary Information. [file 41598_2023_27466_MOESM1_ESM.pdf]

Supplementary information

Luminescent Cd coordination polymer based on Thiazole as a dual-responsive chemosensor for 4-nitroaniline and CrO_4^{2-} in water

*Akram Karbalaee Hosseini, Azadeh Tadjarodi**

Research Laboratory of Inorganic Materials Synthesis, Department of Chemistry, Iran University of Science and Technology (IUST), 16846-13114 Tehran, Iran

Corresponding author: *Tel: +98(21) 77240517; Fax: +98(21) 77491204.*

Email: *tajarodi@iust.ac.ir (A. Tadjarodi)*

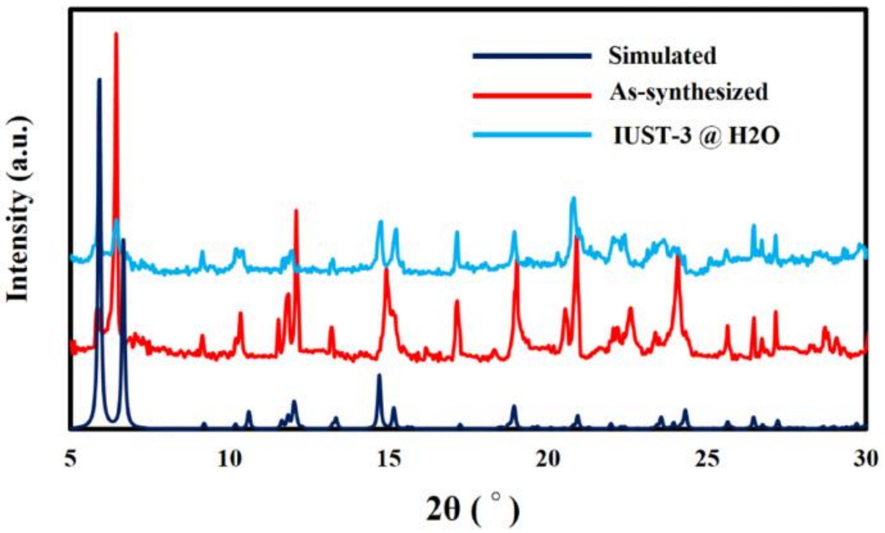

Fig. S1. *XRD patterns of the* ***IUST-3****: Simulated, as-synthesized, immersed in water.*

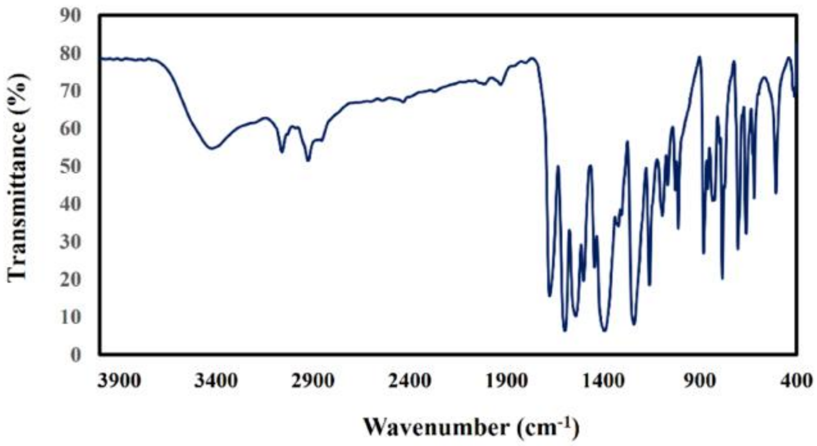

Fig. S2*. FT-IR spectrum of the* ***IUST-3****.*

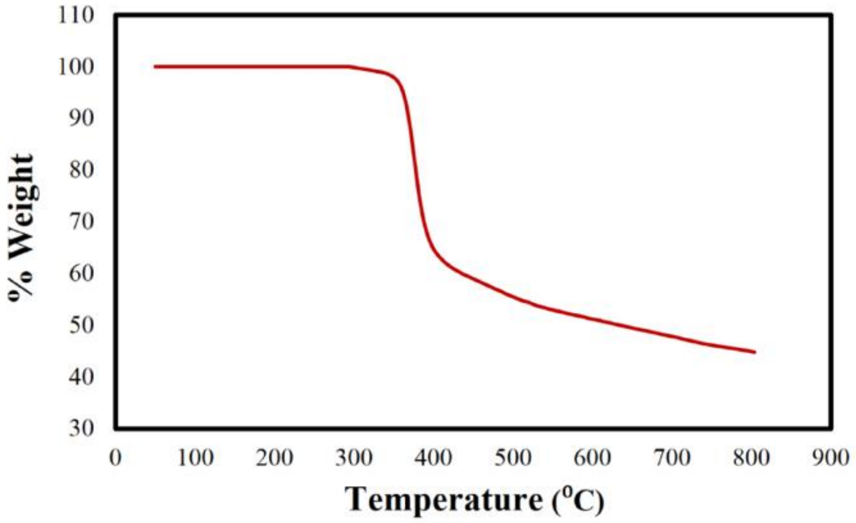

Fig. S3. *TGA curve of the **IUST-3**.*

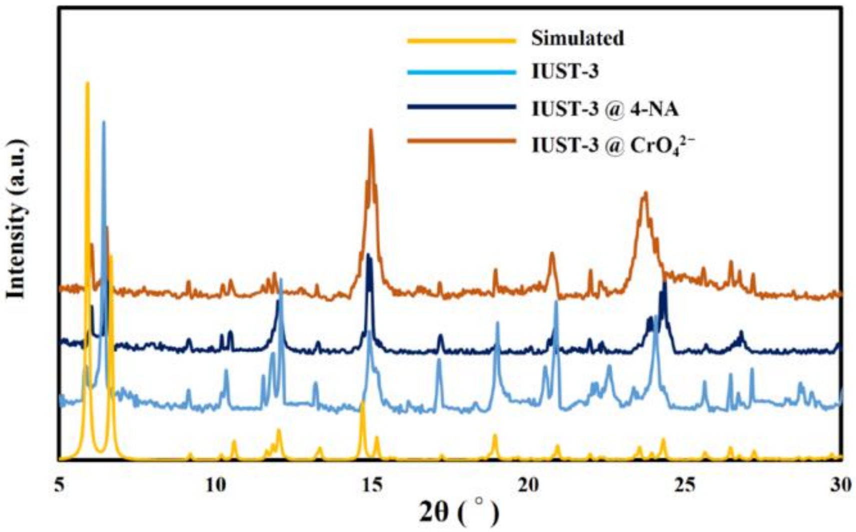

Fig. S4. *XRD patterns of the **IUST-3**: Simulated, as-synthesized, immersed in water solution of 4-NA, immersed in water solution of* CrO_4^{2-} *anion.*

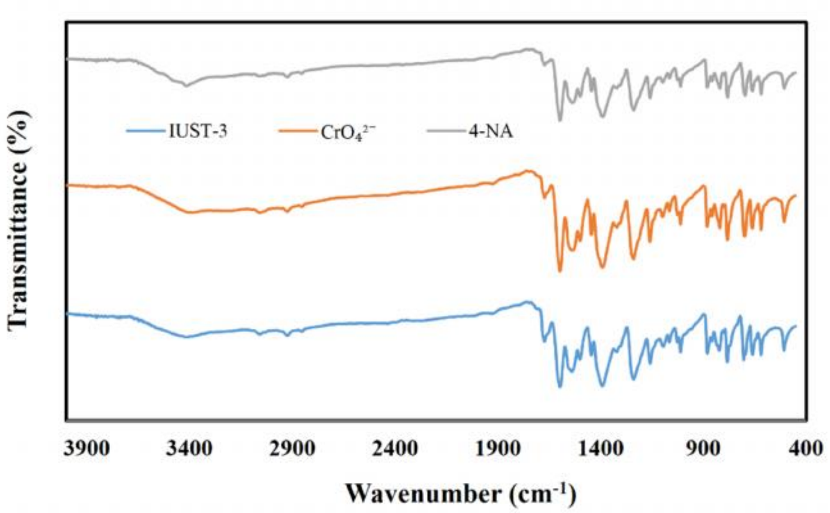

Fig. S5. *FT-IR spectra of the* ***IUST-3****, soaking in 4-NA, and soaking in* CrO_4^{2-}

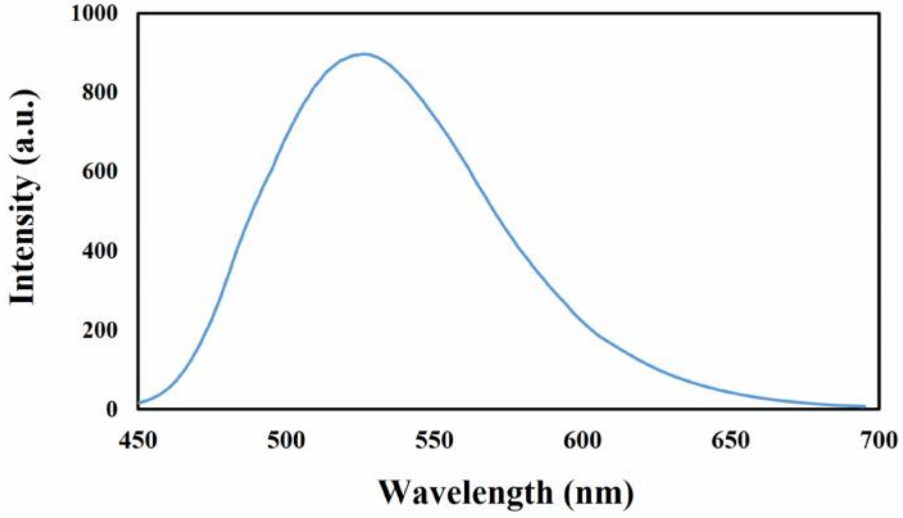

Fig. S6. *The solid-state emission spectrum of the* ***IUST-3*** *at room temperature (*λ_{ex} *=380).*

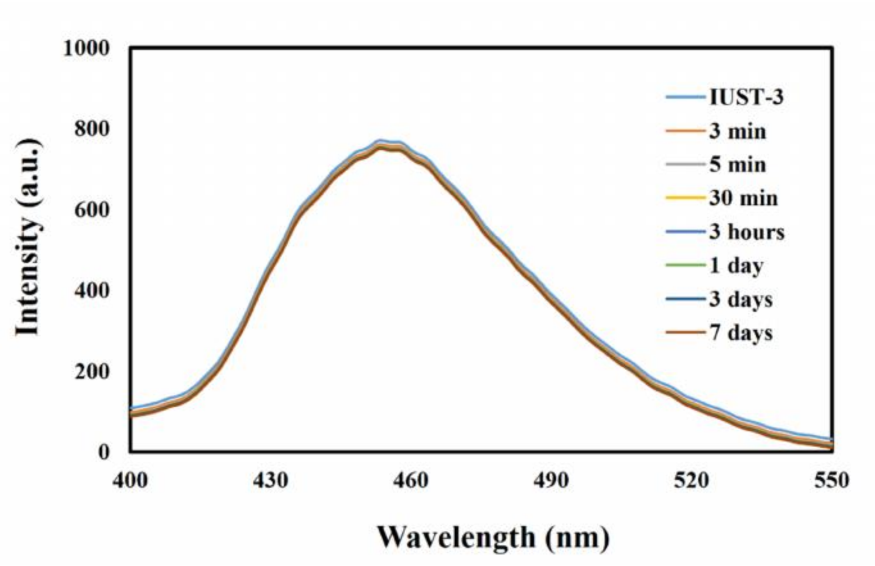

Fig. S7. *The intensity of luminescence change over time for the* ***IUST-3*** *in water.*

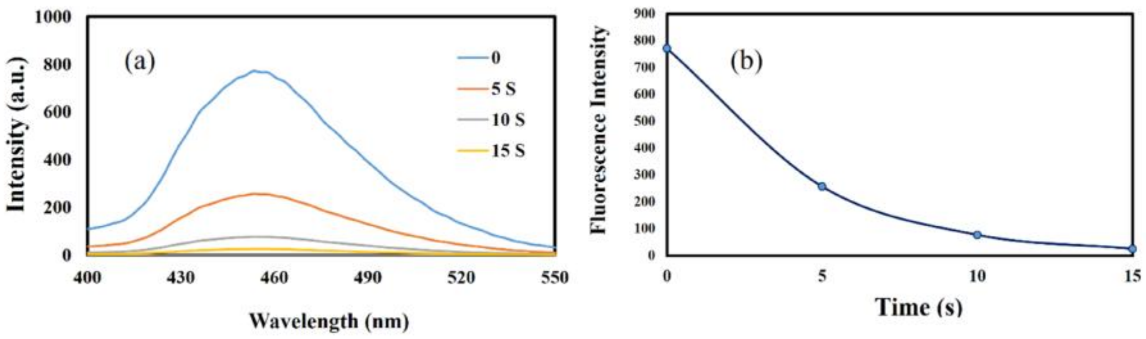

Fig .S8. *(a) Response time florescence spectra of the* ***IUST-3*** *for 4-NA, (b) the time response curve of the* ***IUST-3*** *for 4-NA*

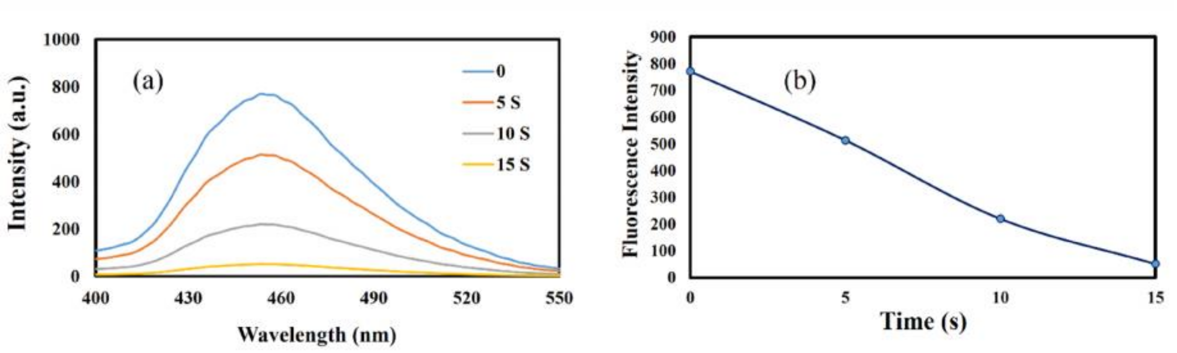

Fig .S9. *(a) Response time florescence spectra of* ***IUST-3*** *for* CrO_4^{2-}*, (b) The time response curve of* ***IUST-3*** *for* CrO_4^{2-}

Table. S1. *Data Collection and refinement parameters for Single Crystal Analysis of the* ***IUST-3****.*

Crystal data	
Chemical formula	$C_{28}H_{16}CdN_4O_5S_2$
M_r	*664.97*
Crystal system, space group	*Triclinic, P*$\bar{1}$
Temperature (K)	*290*
a, b, c (Å)	*9.766 (2), 13.729 (3), 15.495 (3)*
α, β, γ (°)	*102.93 (3), 96.74 (3), 96.05 (3)*
V (Å^3)	*1992.2 (7)*
Z	*2*
Radiation type	*Mo Kα*
μ (mm^{-1})	*0.68*
Crystal size (mm)	*0.3 × 0.25 × 0.2*
Data collection	
Diffractometer	*MAR-345dtb*
Absorption correction	*Multi-scan*<br>*R.H. Blessing, Acta Crystallogr., Sect A 1995, 51, 33-38*
Tmin, Tmax	*0.903, 1.160*
No. of measured, independent and observed [I > 2σ(I)] reflections	*13945, 7232, 5824*
R_{int}	*0.052*
$(\sin \theta/\lambda)_{max}$ (Å^{-1})	*0.625*
Refinement	
$R[F^2 > 2\sigma(F^2)]$, $wR(F^2)$, S	*0.068, 0.218, 1.04*
No. of reflections	*7232*
No. of parameters	*361*
H-atom treatment	*H-atom parameters constrained*
$\Delta\rho_{max}$, $\Delta\rho_{min}$ (e Å^{-3})	*0.75, −1.13*
CCDC number	*2207989*

Table S2. *Comparison of Ksv values and LOD between the **IUST-3** and the selected MOFs for 4-NA.*

LMOFs	LOD (µM)	K_{sv} (M^{-1})	Ref.
$\{[Zn_2(tda)_2(azopy)_2]\cdot DMF\}_n$	0.47	2.75×10^4	[S1]
$\{Zn_4(TPOM)(1,4\text{-}NDC)_4\}n$	0.64	7.87×10^4	[S2]
$[Zn(bpba)(NO_3)]$	0.72	2.28×10^4	[S3]
$[H_3O][Mn_3(CPCP)(CH_3COO)(DMF)]\cdot 11H_2O$	2.04	5.19×10^4	[S4]
$\{[Cd_2(tpbn)(mbhna)_2].2DMF\}n$	12.9	2.87×10^4	[S5]
$\{[Cd(CIP)_2(H2O)_2]n\cdot 2.5H_2O\}$	-	4.59×10^4	[S6]
$Tb_2(TDC)_3(CH_3OH)_2$	-	9.52×10^3	[S7]
$[Cd(H_2BDDA)]n$	4.9	8.79×10^4	[S8]
IUST-3	0.52	1.03×10^5	This work

Table S3. *Comparison of Ksv values and LOD between the **IUST-3** and the selected MOFs for CrO_4^{2-} anion.*

LMOFs	LOD (µM)	K_{sv} (M^{-1})	Ref.
$[Zn(btz)]_n$	10	3.19×10^3	[S9]
$[Zn(ttz)H_2O]_n$	20	2.35×10^3	[S9]
$\{[Cd_3(\mu_4\text{-}cpboda)_2(\mu_{1,1}\text{- }OH_2)_2(phen)_2]\cdot 2DMF\cdot 1.\ 5H_2O\}_n$	4.4	1.43×10^4	[S10]
$[Zn_2(TPOM)(NDC)_2]\cdot 3\cdot 5H_2O$	2.50	0.781×10^4	[S11]
$\{[Eu(L)(HCOO)(H2O)]\}n$	-	1.54×10^3	[S12]
$[Zn(IPA)(L)]n$	13.6	4.34×10^3	[S13]
$[Cd_3(cpota)_2(phen)_3]_n\cdot 5nH2O$	0.418	6.9×10^3	[S14]
$Zn_2(bpdc)_2(tpeb)$	1.07	1.08×10^4	[S15]
IUST-3	1.37	2.93×10^4	This work

References

[S1] Li, L., Zou, J. Y & You, S. Y. A luminescent pillar-layer Zn (II) metal–organic framework for the ultrasensitive detection of nitroaniline. Inorganica Chim. Acta. 509, 119703 (2020).

[S2] Chakraborty, G., Das P. & Mandal, S. K. Strategic construction of highly stable metal–organic frameworks combining both semi-rigid tetrapodal and rigid ditopic linkers: selective and ultrafast sensing of 4-Nitroaniline in water. ACS Appl. Mater. Interfaces.10, 42406-42416 (2018).

[S3] Ji, N. N., Shi, Z. Q., Hu, H. L. & Zheng, H. G. A triphenylamine-functionalized luminescent sensor for efficient p-nitroaniline detection. Dalton Trans Dalton Trans. 47, 7222-7228 (2018).

[S4] Lv, M. X. et al. Manganese cyclotriphosphazene multicarboxylate frameworks and composite encapsulated 1, 3, 6, 8-tetrakis (p-benzoic acid) pyrene as visualization of highly selective fluorescence sensors for aromatic compounds with NH_2/NO_2 Group. Cryst. Growth Des. 21, 6824-6839 (2021).

[S5] Chakraborty, G., Das, P. & Mandal, S. K. Structural diversity in luminescent MOFs containing a bent electron-rich dicarboxylate linker and a flexible capping ligand: selective detection of 4-nitroaniline in water. Chem. Asian J. 14, 3712-3720 (2019).

[S6] Wang, X. Q. et al. A multifunctional 1D Cd-based metal-organic complex for the highly luminescent sensitive detection of Fe^{3+}, $CrO_4^{2-}/Cr_2O_7^{2-}$, and nitroaromatic explosives. J. Solid State Chem. 274, 40-46 (2019).

[S7] Xu, K. et al. Selective fluorescence detection of anilines and Fe^{3+} ions by two lanthanide metal–organic frameworks. RSC Adv. 6, 91741-91747 (2016).

[S8] Shu, T. et al. A new three-dimensional Cd (II) metal-organic framework for highly selective sensing of Fe^{3+} as well as nitroaromatic compounds. ChemistrySelect. 2, 12046-12050 (2017).

[S9] Cao, C. S., Hu, H. C., Xu, H., Qiao, W. Z. & Zhao, B. Two solvent-stable MOFs as a recyclable luminescent probe for detecting dichromate or chromate anions. CrystEngComm. 18, 4445-4451 (2016).

[S10] Yang, D. D., Lu, L. P. & Zhu, M. L. Structural diversity, magnetic property, or luminescence sensing of Co (II) and Cd (II) coordination polymers derived from designed 3, 3′-((5-carboxy-1, 3-phenylene) bis (oxy)) dibenzoic acid. Dalton Trans. 48, 10220-10234 (2019).

[S11] Lv, R. et al.Zinc metal–organic framework for selective detection and differentiation of Fe(III) and Cr(VI) ions in aqueous solution. Inorg. Chem. 56, 12348-12356 (2017).

[S12] Huang, X. H. et al. Two lanthanide metal–organic frameworks as sensitive luminescent sensors for the detection of Cr^{2+} and $Cr_2O_7^{2-}$ in aqueous solutions. CrystEngComm. 20, 189-197 (2017).

[S13] Parmar, B., Rachuri, Y., Bisht, K. K., Laiya, R. & Suresh, E. Mechanochemical and conventional synthesis of Zn(II)/Cd(II) luminescent coordination polymers: dual sensing probe for selective detection of chromate anions and TNP in aqueous phase. Inorg. Chem. 56, 2627-2638 (2017).

[S14] Li, S., Lu, L., Zhu, M., Yuan, C. & Feng, S. A bifunctional chemosensor for detection of volatile ketone or hexavalent chromate anions in aqueous solution based on a Cd (II) metal–organic framework. Sens. Actuators B Chem. 258, 970-980 (2018).

[S15] Rath, B. B. & Vittal, J. J. Water stable Zn (II) metal–organic framework as a selective and sensitive luminescent probe for Fe(III) and chromate ions. Inorg. Chem. 59, 8818-8826 (2020).
